# SRSF3 Knockdown Inhibits Lipopolysaccharide-Induced Inflammatory Response in Macrophages

**DOI:** 10.3390/cimb46060372

**Published:** 2024-06-20

**Authors:** Yu Fu, Yanjing Wang, Luyao Zhang, Tianliu He, Weiye Shi, Xueling Guo, Yingze Wang

**Affiliations:** College of Food Science and Biology, Hebei University of Science and Technology, Shijiazhuang 050018, China; 13323040621@163.com (Y.W.); 13171672345@163.com (L.Z.); 15028146761@163.com (T.H.); feiwudeyezi@126.com (W.S.); xlguo1989@163.com (X.G.)

**Keywords:** SRSF3, inflammation, macrophages, alternative splicing, MD2

## Abstract

Serine/arginine-rich splicing factor 3 (SRSF3), the smallest member of the SR protein family, serves multiple roles in RNA processing, including splicing, translation, and stability. Recent studies have shown that SRSF3 is implicated in several inflammatory diseases. However, its impact on macrophage inflammation remains unclear. Herein, we determined the expression of SRSF3 in inflammatory macrophages and found that the level of SRSF3 was increased in macrophages within atherosclerotic plaques, as well as in RAW-264.7 macrophages stimulated by lipopolysaccharides. Moreover, the downregulation of SRSF3 suppressed the levels of inflammatory cytokines by deactivating the nuclear factor κB (NFκB) pathway. Furthermore, the alternative splicing of myeloid differentiation protein 2 (MD2), a co-receptor of toll-like receptor 4 (TLR4), is regulated by SRSF3. The depletion of SRSF3 increased the level of the shorter MD2B splicing variants, which contributed to inflammatory inhibition in macrophages. In conclusion, our findings imply that SRSF3 regulates lipopolysaccharide-stimulated inflammation, in part by controlling the alternative splicing of MD2 mRNA in macrophages.

## 1. Introduction

SRSF3 is a member of the highly conserved SR protein family, which plays essential roles in regulating alternative and constitutive splicing [1]. Studies have highlighted the multifaceted involvement of SRSF3 in various steps of mRNA metabolism, including splicing, translation, and stability [2]. SRSF3 is widely expressed in various tissues. However, the dysfunctional expression or improper regulation of SRSF3 has been associated with several diseases, including cancer and neurological and cardiac disorders [3,4,5]. Interestingly, SRSF3 has been related to the occurrence of inflammatory disorders. The degradation of SRSF3 in the liver contributes to hepatic steatosis, fibrosis, and inflammation [6]. SRSF3 inhibits the translation of highly upregulated immune genes in activated microglia, which are immune cell lines in the brain [7]. However, the role of SRSF3 in inflammation is still unclarified.

Inflammation is a vital immune reaction that serves as the body’s defense mechanism against infection, injury, or irritation. However, chronic or excessive inflammation may lead to various disorders, including arthritis, cancer, and atherosclerosis [8,9,10]. Atherosclerosis, a chronic inflammatory disease, involves macrophages as the primary inflammatory cell line in atherosclerotic lesions [11,12,13,14,15]. Macrophages regulate the inflammatory response by promptly responding to stimuli and releasing a multitude of pro-inflammatory factors, including TNFα, iNOS, and COX2 [16]. Therefore, we investigated SRSF3 expression in atherosclerotic lesions and its regulation of the inflammatory response in macrophages.

Toll-like receptor (TLR) pathways are the main inflammatory signaling pathways in macrophages, with TLR4 inducing myeloid differentiation 88 (MyD88)-dependent signaling and NFκB pathway activation upon lipopolysaccharide stimulation [17,18]. Notably, alterations in mRNA alternative splicing within the TLR4 signal transduction can negatively regulate the inflammatory response [19,20]. For instance, MD2, a co-receptor of TLR4, can exist in two spliced forms (MD2 and MD2B), which exhibit contradictory functions in macrophages. Moreover, the shorter MD2B splicing isoform acts as a dominant negative inhibitor of the TLR4–NFκB axis [21,22]. Nevertheless, the exact functions and underlying mechanisms through which splicing factors, such as SRSF3, regulate macrophage inflammation are still unclear.

We observed that SRSF3 was highly expressed in macrophages within atherosclerotic plaques and was upregulated in pro-inflammatory macrophages when exposed to lipopolysaccharides. Furthermore, we observed that the downregulation of SRSF3 elevated the mRNA levels of MD2B, leading to the inhibition of inflammation mediated by NFκB in RAW-264.7 macrophages. Taken together, our findings indicated that SRSF3 regulated the lipopolysaccharide-induced inflammatory response in macrophages by modulating TLR4 signaling. We also found that this mechanism was partly responsible for regulating the alternative splicing of MD2 mRNA via SRSF3.

## 2. Materials and Methods

### 2.1. ApoE^−/−^ Mouse Model for Atherosclerosis Analysis

ApoE^−/−^ mice (male, 8 weeks old) were acquired from Vital River Laboratory Animal Technology (Beijing, China). To induce atherosclerosis, ApoE^−/−^ mice were fed a high-fat diet (HFD) containing 1.25% cholesterol and 20% fat. The control ApoE^−/−^ mice were given a standard chow diet (NC). All animal procedures were approved by the Institutional Animal Care and Use Committee of Hebei University of Science and Technology, following their guidelines, with the ethical review number H2020208002. The heart, along with the upper aortic root, was fixed and embedded at optimal cutting temperature (Sakura Finetek, Torrance, CA, USA). Serial aortic root sections, measuring 7 μm in thickness, were made from the point where the aortic valve is visible. These cryosections were stained with Oil Red O and hematoxylin and eosin (Beyotime, Shanghai, China) to evaluate the atherosclerotic lesions in the aortic sinus cross-sections.

### 2.2. Immunohistochemical and Immunofluorescence Staining

The serial aortic sinus cross-sections were dehydrated and embedded in paraffin. Following deparaffinization and rehydration, the sections were blocked with BSA (Solarbio, Beijing, China) for 30 min. Subsequently, the sections were exposed to an SRSF3 antibody (1:100, Abcam, Cambridge, UK) overnight at 4 °C, and then with secondary antibodies for 1 h. The images were captured using a Leica Q550cw Graphic Analysis System (Leica Microsystems, Wetzlar, Germany).

For the immunofluorescence double staining assay of aortic sinus cross-sections, primary antibodies against SRSF3 (1:100, Abcam) and CD68 (1:100, Abcam) were used and incubated at 4 °C overnight. The sections were then labeled with appropriate secondary antibodies for 30 min, followed by nuclear staining with DAPI (ThermoFisher Scientific, Waltham, MA, USA) for 10 min. The images were obtained using a laser scanning confocal microscope (Olympus, Tokyo, Japan).

### 2.3. Cell Culture

The RAW-264.7 macrophages supplied by the National Collection of Authenticated Cell Cultures (Shanghai, China), and subsequently cultured in DMEM (Hyclone, Logan, UT, USA) containing 10% FBS (ThermoFisher Scientific).

### 2.4. Transfection and RNA Interference

The pECMV-FLAG-SRSF3 plasmid was transfected using Lipofectamine 2000 (Invitrogen, Carlsbad, CA, USA). The siRNA transfection was carried out using Lipofectamine RNAi MAX (Invitrogen). The siRNA synthesis was performed by GenePharma (Shanghai, China). The sequence of the mouse Srsf3 siRNAs was 5′-GAGUGGAACUGUCGAAUGG-3′, 5′-CCGUCUCGAUCCUUCUCUA-3′. The sequence of the control siRNA was 5′-UUCUCCGAACGUGUCACGU-3′.

### 2.5. RT-qPCR Analysis 

The RAW-264.7 cell line was grown in serum-free DMEM with 1 μg/mL lipopolysaccharide (Sigma-Aldrich, St. Louis, MO, USA) for 3 h. The cell line was then collected using TransZol Up (TransGen, Beijing, China). The total RNA was isolated with a TransZol Up Plus RNA Kit (TransGen), and the reverse transcription was performed using the reverse transcription kit (TransGen). The qPCR with SYBR (TransGen) detection was performed on the QuantStudio 3 Real-Time PCR System (Applied Biosystems, Waltham, MA, USA). The separation of the RT-PCR products was achieved using 8% non-denaturing PAGE. The images were captured using the ChemicDoc XRS+ system (Bio-Rad, Hercules, CA, USA), followed by an analysis with ImageJ software (version 1.46r). The sequences of all primers are listed in Table 1.

### 2.6. Immunoblotting

The total protein samples were isolated with RIPA buffer. The method was performed as described previously [23]. The primary antibodies included rabbit anti-SRSF3 (1:1000, Abcam), mouse anti-β-actin (1:5000, Proteintech, Rosemont, IL, USA), rabbit anti-COX2 (1:1000, Abcam), rabbit anti-iNOS (1:1000, Abcam), mouse anti-NFκB p65 (1:1000, Proteintech), rabbit anti-phospho-NFκB p65 (1:1000, Bioworld, Visalia, CA, USA), rabbit anti-IκBα (1:1000, Proteintech), and rabbit anti-phospho-IκBα (1:1000, Bioworld) antibodies. The quantification of the protein blots was achieved with ImageJ software.

### 2.7. ELISA

The RAW-264.7 cell line was exposed to 1 μg/mL lipopolysaccharides for 12 h. The IL6 and TNFα levels in the culture medium were detected with commercial ELISA kits (ExCell Bio, Suzhou, China).

### 2.8. Statistical Analysis

The data are represented by means ± SEMs. The statistical differences were compared through a one-way ANOVA or Student’s *t*-test (GraphPad-Prism 9). Here, *p* < 0.05 was utilized to indicate statistical significance.

## 3. Results

### 3.1. SRSF3 Is Highly Expressed in the Macrophages of Atherosclerotic Plaques

**Figure 1:**
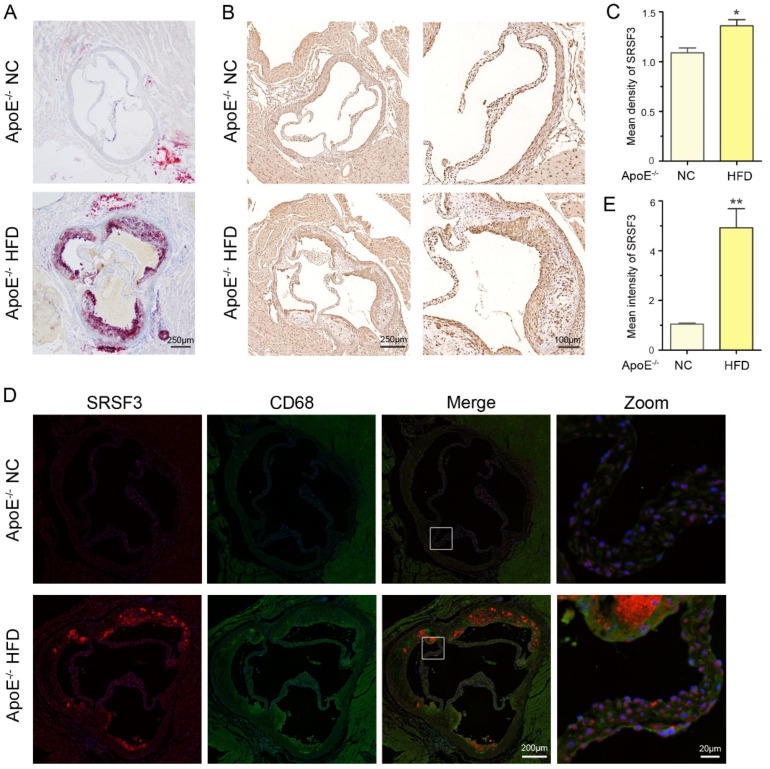
SRSF3 upregulation in ApoE^−/−^ mouse model and macrophages. (**A**) Oil Red O staining was conducted to visualize the burden of atherosclerosis in the aorta and aortic roots of ApoE^−/−^ mice with HFD treatment. (**B**,**C**) Immunohistochemical staining was employed to examine SRSF3 expression in plaque sample from ApoE^−/−^ mice. Scale bar = 250/100 μm. Mean optical density of SRSF3 protein was analyzed (*n* = 3, * *p* < 0.05 versus ApoE^−/−^ NC mice). (**D**,**E**) Double immunofluorescence staining for SRSF3 (red) and CD68 (green) was performed in the plaque samples of ApoE^−/−^ mice. Scale bar = 200/20 μm. Mean fluorescence intensity of the SRSF3 protein was analyzed (*n* = 3. ** *p* < 0.01 versus ApoE^−/−^ NC mice).

Atherosclerosis is a lipid-regulated chronic inflammatory disorder. In addition to foam cell formation, inflammatory macrophages play a vital role in the occurrence of atherosclerotic plaques. To investigate the potential role of SRSF3 in inflammatory macrophages, we examined its expression in ApoE^−/−^ mice, a widely used model for atherosclerosis. The mice were divided into groups and fed an NC or HFD for 8 weeks. The Oil Red O staining of the aorta and aortic root confirmed the formation of atherosclerotic plaque (Figure 1A). We then explored the SRSF3 protein expression in these atherosclerotic lesions. Both the immunohistochemical and immunofluorescence staining measurements revealed increased SRSF3 protein levels in the atherosclerotic plaques (Figure 1B–E). Furthermore, the double immunofluorescence staining for SRSF3 and CD68 (a macrophage biomarker) indicated that the SRSF3 expression was predominantly situated in macrophages within the atherosclerotic lesion (Figure 1D,E). The upregulation of SRSF3 in these lesions implies that SRSF3 is responsible for the inflammatory response of macrophages.

### 3.2. The Expression of SRSF3 Is Upregulated in Lipopolysaccharide-Stimulated RAW-264.7 Cell Line

**Figure 2:**
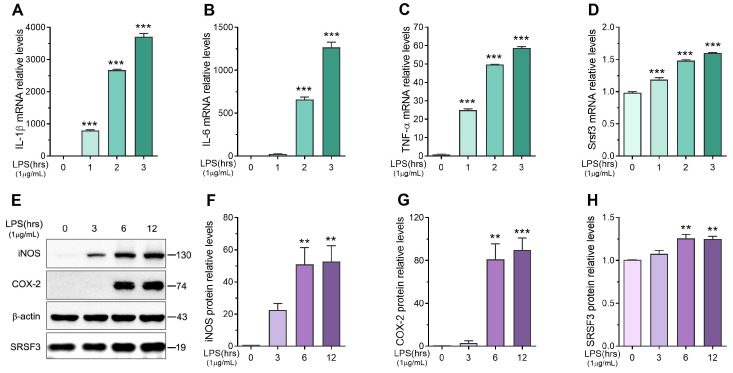
The expression level of SRSF3 was increased in RAW-264.7 cell lines treated with lipopolysaccharide: (**A**–**C**) the inflammatory response of the macrophages was detected by measuring the mRNA expression of cytokines, including IL1β, IL6, and TNFα; (**D**) the level of SRSF3 in the lipopolysaccharide-treated RAW-264.7 cell line was determined using qPCR, with GAPDH as the housekeeping gene; (**E**–**H**) the protein levels of iNOS, COX2, and SRSF3 were assessed using immunoblotting with β-actin as an internal control. Mean ± SEM (*n* = 3). Statistical differences compared to controls (timepoint 0), ** *p* < 0.01, *** *p* < 0.001.

To investigate the role of SRSF3 in inflammation in vitro, we detected the expression of SRSF3 in RAW-264.7 cell lines exposed to lipopolysaccharides. Initially, we determined the mRNA and protein levels of lipopolysaccharide-stimulated inflammatory cytokines via qPCR and immunoblotting. The data showed that the mRNA levels of IL1β, IL6, and TNFα (Figure 2A–C), as well as the protein levels of iNOS and COX2 (Figure 2E–G), increased in a time-dependent fashion following lipopolysaccharide stimulation. Simultaneously, we found an obvious increase in SRSF3 mRNA expression following lipopolysaccharide exposure (Figure 2D). Additionally, the enhancement in SRSF3 protein expression was confirmed through immunoblotting, as shown in Figure 2E,H. These findings indicated that the mRNA and protein levels of SRSF3 were elevated in the lipopolysaccharide-stimulated RAW-264.7 cell line, implying its potential involvement in regulating the inflammatory response of macrophages.

### 3.3. Inhibition of SRSF3 Downregulates Lipopolysaccharide-Induced Expression of Inflammatory Cytokines in RAW-264.7 Cell Line

**Figure 3:**
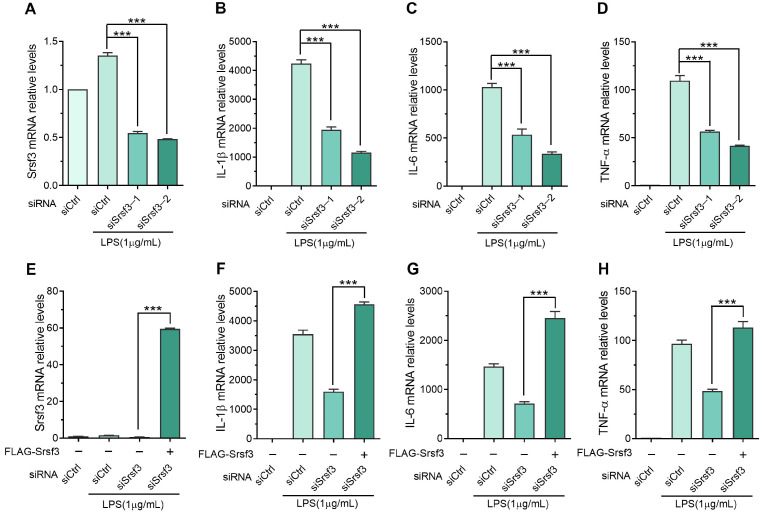
SRSF3 depletion downregulated mRNA levels of cytokines in lipopolysaccharide-treated RAW-264.7 cell line: (**A**–**D**) RAW-264.7 cell line was transfected with siRNAs, followed by treatment with and without 1 μg/mL lipopolysaccharides for 3 h; (**E**–**H**) RAW-264.7 cell line underwent co-transfection with siRNAs and pECMV-FLAG-Srsf3 plasmids. The mRNA expression of SRSF3 and cytokines was evaluated via qPCR. Note: siCtrl, negative control siRNA; siSrsf3-1/2, Srsf3 siRNA-1/2; siSrsf3, Srsf3 siRNA-1 and -2. Mean ± SEM (*n* = 3). Statistical differences compared to lipopolysaccharide-treated siSrsf3, *** *p* < 0.001.

**Figure 4:**
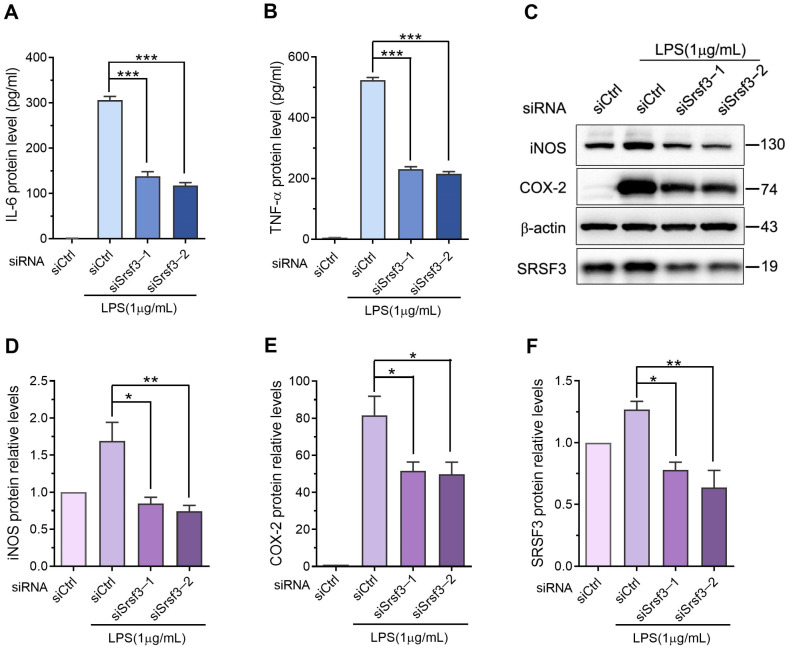
SRSF3 depletion downregulated the protein levels of cytokines in lipopolysaccharide-treated RAW-264.7 cell line: (**A**,**B**) RAW-264.7 cell line was transfected with siRNAs, followed by treatment with and without 1 μg/mL lipopolysaccharide for 12 h, whereby the levels of IL6 and TNFα were detected through an ELISA; (**C**–**F**) the cell line was collected and the levels of iNOS and COX2 were assessed via immunoblotting, using β-actin as an internal control. Note: siCtrl, negative control siRNA; siSrsf3-1/2, Srsf3 siRNA-1/2. Mean ± SEM (*n* = 3). Statistical differences compared to lipopolysaccharide-treated siCtrl, * *p* < 0.05, ** *p* < 0.01, *** *p* < 0.001.

To explore the role of SRSF3 in the lipopolysaccharide-induced inflammatory response of macrophages, SRSF3-specific siRNAs were used to downregulate the SRSF3 expression. The results shown in Figure 3A–D demonstrate that the mRNA levels of IL1β, IL6, and TNFα were upregulated upon lipopolysaccharide stimulation but significantly decreased following SRSF3 knockdown. In addition, our study indicates that the overexpression of SRSF3 could rescue the downregulation of lipopolysaccharide-stimulated cytokines through SRSF3 siRNAs (Figure 3E–H). Furthermore, ELISA and immunoblotting measurements were conducted to examine the protein levels of lipopolysaccharide-induced inflammatory cytokines after SRSF3 knockdown. The downregulation of SRSF3 markedly reduced the expression of lipopolysaccharide-induced IL6 and TNFα proteins in RAW-264.7 macrophages (Figure 4A,B). Additionally, as shown in Figure 4C–F, SRSF3 knockdown led to a downregulation of the lipopolysaccharide-stimulated protein levels of iNOS and COX2. These results indicated that the downregulation of SRSF3 could inhibit the lipopolysaccharide-induced expression of inflammatory cytokines in the RAW-264.7 cell line.

### 3.4. Knockdown of SRSF3 Downregulates Lipopolysaccharide-Stimulated NFκB Signaling Pathway Activation in RAW-264.7 Cell Line

**Figure 5:**
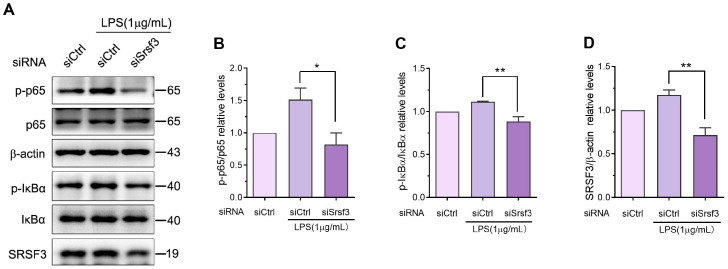
SRSF3 knockdown reduced lipopolysaccharide-stimulated NFκB pathway activation in RAW-264.7 cell line. (**A**) RAW-264.7 cell line underwent transfection with SRSF3 siRNAs or siCtrl, followed by treatment with 1 μg/mL lipopolysaccharide for 1 h. The phosphorylated levels of p65 and IκBα in the NFκB axis were determined via immunoblotting. (**B**–**D**) The phosphorylated p65 and IκBα protein levels were normalized to p65 and IκBα, respectively, while the SRSF3 protein level was normalized to β-actin. Note: siCtrl, negative control siRNA; siSrsf3, Srsf3 siRNA-1 and -2. Mean ± SEM (*n* = 3). Statistical differences compared to lipopolysaccharide-treated siCtrl, * *p* < 0.05, ** *p* < 0.01.

Lipopolysaccharides activate TLR4 on the surfaces of macrophages, triggering downstream signaling cascades that lead to NFκB phosphorylation and activation. NFκB-active molecules subsequently translocate to the nucleus and promote the transcription and production of various pro-inflammatory cytokines. To explore the involvement of SRSF3 in lipopolysaccharide-stimulated NFκB pathway activation, we analyzed the phosphorylation levels of p65 and IκBα via immunoblotting after SRSF3 depletion using siRNA. As shown in Figure 5A–C, the phosphorylation levels of p65 and IκBα were markedly elevated following exposure to lipopolysaccharide for 1 h, suggesting activation of the NFκB axis. However, when SRSF3 was knocked down, the protein levels of phosphorylated p65 and phosphorylated IκBα were remarkably reduced compared to the control cell line exposed to lipopolysaccharide (Figure 5A,D). These findings suggested that SRSF3 knockdown led to decreased NFκB activation in the lipopolysaccharide-stimulated RAW-264.7 cell line. Thus, our findings indicated that SRSF3 played a pivotal role in the expression of inflammatory cytokines in macrophages through the NFκB axis.

### 3.5. SRSF3 Regulates the Alternative Splicing of MD2 in Lipopolysaccharide-Stimulated RAW-264.7 Cell Line

**Figure 6:**
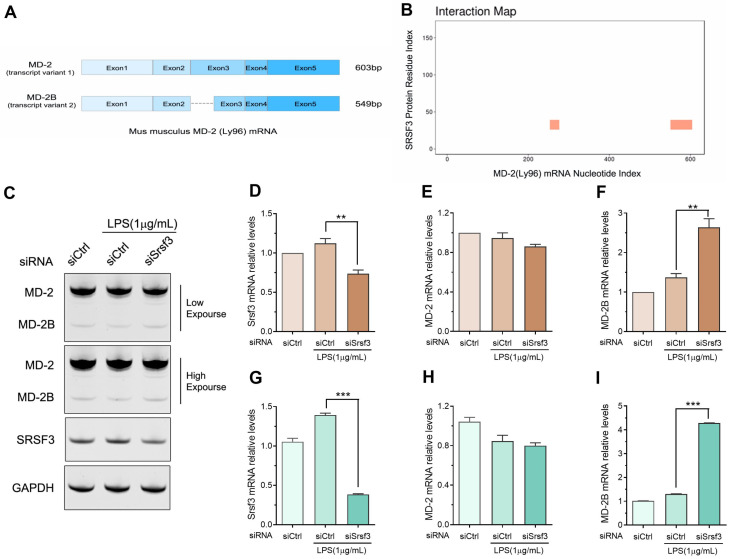
SRSF3 regulated the alternative splicing of MD2 mRNA. RAW-264.7 cell line underwent transfection with SRSF3 siRNAs or siCtrl, followed by treatment with 1 μg/mL lipopolysaccharides for 3 h. (**A**) The sequence alignment was performed to distinguish between the two transcript variants of MD2(Ly96) mRNA. (**B**) The interaction map of SRSF3 protein and MD2(Ly96) mRNA was generated using catRAPID omics v2.0. (**C**–**F**) RT-qPCR was employed for product- amplification from both MD2 and MD2B, with quantitation analysis results shown in (**D**) SRSF3, (**E**) MD2, and (**F**) MD2B. (**G**–**I**) The mRNA expression levels of SRSF3, MD2, and MD2B were assessed via qPCR. Statistical differences compared to lipopolysaccharide-treated siCtrl, ** *p* < 0.01, *** *p* < 0.001.

MD2, also known as lymphocyte antigen 96 (Ly96), functions as a co-receptor of lipopolysaccharide-mediated TLR4 signaling [24,25]. The mouse MD2 produced two splicing variants, namely MD2 and MD2B (Figure 6A). The shorter MD2B variant is missing the first 54 bases of exon 3, which hinders the expression of TLR4 on the cell surface and competitively suppresses the lipopolysaccharide-induced NFκB activation [21]. Using catRAPID omics [26], we predicted a potential interaction between SRSF3 and MD2 mRNA. As shown in Figure 6B, this interaction may occur at sites within exons 3 and 5 of MD2(Ly96) mRNA. Notably, the predicted binding site within exon 3 is present in MD2 mRNA but absent in MD2B mRNA. These data suggest that the SRSF3 protein potentially interacted with MD2 mRNA and regulated the alternative splicing of MD2.

To further investigate the impact of SRSF3 expression on MD2 splicing, we employed semi-quantitative PCR and qPCR tests to measure the levels of pro-inflammatory MD2 and anti-inflammatory MD2B splicing variants. Our results showed that the downregulation of SRSF3 had a minimal effect on the expression of the MD2 splicing variant but resulted in an increase in the mRNA level of the MD2B splicing variant (Figure 6C–I). These observations demonstrated that SRSF3 played a crucial role in modulating inflammatory cytokine expression via the alternative splicing of MD2 in RAW-264.7 macrophages.

## 4. Discussion

Macrophages are vital cell lines of the immune system that play a key role in modulating inflammation. They respond rapidly to triggers and produce numerous inflammatory cytokines, which can contribute to the occurrence of diverse tumors, infectious diseases, autoimmune diseases, and cardiovascular diseases [27,28,29]. Therefore, the proper regulation of the macrophage inflammatory response is crucial for disease prevention and treatment [30]. It should be noted that RNA-binding proteins (RBPs) are essential post-transcriptional mediators of macrophage inflammation [31]. Specifically, the regulation of RBPs can suppress the inflammatory response during the progression of inflammatory diseases [32]. Exploring the regulatory mechanisms of RBPs in inflammatory factors and macrophage inflammatory responses is of great significance for understanding, preventing, and treating inflammatory diseases.

SRSF3 belongs to the SR protein family, which contains conserved splicing factors found in all metazoans and plants. SRSF3 has multifunctional activities, affecting various cellular processes, including alternative splicing, mRNA export, decay, and translation. The dysregulation of SRSF3, leading to the altered splicing of specific genes, has been linked to many human disorders such as cancer, neurological disorders, and inflammatory diseases [33]. Herein, we observed that SRSF3 is highly expressed in macrophages of atherosclerotic plaques and upregulated in lipopolysaccharide-stimulated macrophages. Additionally, we found that the inhibition of SRSF3 downregulated the expression of inflammatory cytokines and the NFκB pathway activation induced by lipopolysaccharides. These observations imply that SRSF3 is responsible for the modulation of inflammatory macrophages. However, additional investigations are warranted to understand the direct impact of SRSF3 on atherosclerosis and other inflammatory diseases.

Researchers have indicated that the process of alternative splicing within genes is linked to TLR4 pathway activation [34,35]. MD2B, a spliced variant of MD2 that lacks part of exon 3, can function as a negative regulator of the TLR4–NFκB axis [21,22]. Herein, the splicing analysis revealed that the expression of the MD2 alternative splice variant (MD2B) was upregulated in SRSF3-depleted macrophages compared to the control group after lipopolysaccharide stimulation. Previous studies have shown that MD2B competitively inhibits the binding of MD2 to TLR4 and fails to activate the NFκB signaling pathway [22]. Our findings suggest that SRSF3 might affect the lipopolysaccharide-induced inflammatory response of macrophages and the activation of NFκB pathways by regulating the alternative splicing of MD2. The potential for SRSF3 to regulate alternative splicing in other inflammatory genes, and its impact on the overall inflammatory landscape, require further investigation through a high-throughput sequencing analysis.

## 5. Conclusions

In summary, we report the role of SRSF3 in modulating the inflammatory response. SRSF3 exhibits high expression levels in macrophages within atherosclerotic plaques and is upregulated in lipopolysaccharide-stimulated macrophages. Additionally, we found that reducing the SRSF3 levels enhances the mRNA expression of MD2B, leading to the suppression of NFκB-regulated inflammation in RAW-264.7 macrophages. These discoveries uncover the complex mechanisms governing the modulation of inflammation in macrophages.

## Figures and Tables

**Table 1:** Primers for RT-qPCR and RT-PCR.

Mouse Gene	Forward Primers (5′-3′)	Reverse Primers (5′-3′)
*SRSF3*	CTCCTGGCTTTGCTTTCGTC	CCCACGATTCCGACTTCTCT
*GAPDH*	CAGCCTCGTCCCGTAGACA	CGCTCCTGGAAGATGGTGAT
*IL1* *β*	CCCTGCAGCTGGAGAGTGTGGA	TGTGCTCTGCTTGTGAGGTGCTG
*IL6*	CTGCAAGAGACTTCCATCCAGTT	GAAGTAGGGAAGGCCGTGG
*TNF* *α*	ATGAGCACAGAAAGCATGATC	TACAGGCTTGTCACTCGAATT
*MD2 variant*	TTGTGCATGTTGAGTTCATTCCAAGAGGAAAC	CCTTACGCTTCGGCAACTCTATGGAGTTGAC
*MD2B variant*	GATTTGTGCATGTTGAGTTCATTCCAAAGTTGCC	CCCTCGAAAGAGAATGGTATTGATGTATTCACAGTC
*MD2* (RT-PCR)	TGGTTCTGCAACTCCTCCGA	GCAACACATCTGTAATGGCCC

## Data Availability

Data will be made available on request.
